# Cluster of Ebola Virus Disease, Bong and Montserrado Counties, Liberia

**DOI:** 10.3201/eid2107.150511

**Published:** 2015-07

**Authors:** Tolbert G. Nyenswah, Mosaka Fallah, Geoffrey M. Calvert, Stanley Duwor, E. Dutch Hamilton, Vishwesh Mokashi, Sampson Arzoaquoi, Emmanuel Dweh, Ryan Burbach, Diane Dlouhy, John E. Oeltmann, Patrick K. Moonan

**Affiliations:** Ministry of Health and Social Welfare, Monrovia, Liberia (T.G. Nyenswah, M. Fallah, S. Duwor);; Centers for Disease Control and Prevention, Atlanta, Georgia, USA (G.M. Calvert, D. Dlouhy, J.E. Oeltmann, P.K. Moonan);; United Nations International Children’s Emergency Fund, Monrovia (E.D. Hamilton);; United States Navy, Silver Spring, Maryland, USA (V. Mokashi);; Ministry of Health and Social Welfare–Bong County, Suokoko, Liberia (S. Arzoaquoi, E. Dweh);; International Medical Corps, Los Angeles, California, USA (R. Burbach)

**Keywords:** Ebola, contact tracing, trust, Liberia, Ebola virus disease, viruses, cluster investigation

## Abstract

Lack of trust in government-supported services after the death of a health care worker with symptoms of Ebola resulted in ongoing Ebola transmission in 2 Liberia counties. Ebola transmission was facilitated by attempts to avoid cremation of the deceased patient and delays in identifying and monitoring contacts.

Reports of what has become the largest and longest epidemic of Ebola virus disease (EVD) began in March 2014 in West Africa (*1*). To interrupt Ebola transmission, health care authorities must promptly isolate and treat persons with EVD and identify and monitor exposed persons before symptoms develop (*2*). Effective contact tracing can limit the number of new cases; however, a single missed contact can result in many new cases (*3*). Gaps in contact tracing have been reported as challenges for infectious diseases such as sexually transmitted infections and tuberculosis (*4*–*6*). Because contact tracing requires patients to reveal names of persons with whom they have had contact and whom they may have exposed to illness, public health officials must quickly establish trust with sick persons and those at risk for disease (*3*,*7*). 

We describe a cluster of EVD cases involving transmission across 2 jurisdictions in Liberia. Data for this report were derived from interviews, case reporting forms, treatment records, and laboratory results. This EVD cluster highlights the challenges associated with public health measures to interrupt transmission of Ebola. 

## The Investigation

On December 8, 2014, a 78-year-old man (patient 1) from Gbarnga (Bong County), Liberia, was admitted to the Bong County Ebola Treatment Unit (ETU), where test results were positive for Ebola by reverse transcription PCR. He reported recent travel to Monrovia (Montserrado County), where he cared for his 32-year-old son, a health care worker who died from an acute illness.

On December 9, another son of patient 1 (patient 2, 39 years of age), who lived in Monrovia, had fever, headache, and malaise and sought care at hospital A in Bong County. He did not report contact with patient 1, nor did he report that he provided care for his sick brother in Monrovia. On December 10, hematemesis developed, and the patient was transferred to the Bong County ETU and treated for laboratory-confirmed EVD. Contact tracing identified 20 contacts living in Gbarnga. All contacts were initially symptom free and were quarantined at a local holding center for 21-day monitoring. No contacts in Monrovia were reported by patients 1 or 2.

On December 16, Bong County health officials were notified that a 15-year-old girl (patient 3) with fever, subconjunctival hemorrhage, and thrush was at hospital A. She had traveled 4 hours by taxi from Monrovia to be near her ill grandfather and father (patients 1 and 2) and did not report exposure to EVD patients or contacts in Monrovia. She was admitted to the ETU, and EVD was confirmed. 

The next day, 4 additional family members who traveled by taxi from Monrovia were stopped at a roadside monitoring station in Gbarnga. All had fever and nonhemorrhagic symptoms and were transferred by ambulance to the ETU for evaluation; 2 family members (patients 4 and 5) had positive test results for EVD. The 2 family members whose results were negative for EVD, along with the taxi driver and a nonfamilial passenger, were transferred to a local holding center for 21-day monitoring. Contact investigations for patients 4 and 5 revealed no new contacts in Monrovia, but the patients reported that they resided in the same house in Monrovia with patients 2 and 3, who were receiving treatment in Bong County. Because family members with EVD had recently arrived from Monrovia and were being treated in Bong County, yet sources of infection and additional contacts were uncertain, Bong County requested that Montserrado County health officials conduct an investigation to identify patients and contacts at the Monrovia address so that potential EVD patients could be isolated and monitored. 

The Monrovia investigation revealed that patients 1–3 had contact with patient 1’s ill son, who was designated the putative source-patient (patient 0). Patient 0 was a nurse’s aide at a community clinic. Fever, headache, joint pain, and abdominal pain developed in patient 0 on November 14, 2014, and he was cared for at home by his family for 7 days while his symptoms worsened. Although the patient and family members were aware of the EVD epidemic, they did not think patient 0 had EVD because he had no vomiting, diarrhea, and hemorrhagic symptoms; they believed he had a spiritual illness. On November 21, he was taken to a church with the hope that he would be healed through prayer. He died there on November 24, and his body was carried to his residence for mourning and burial preparation. Because all unexplained deaths were presumed to be Ebola related, an EVD burial crew retrieved his body for cremation the following day, despite resistance from the family and only after persuasion by local community leaders. No postmortem specimen was collected for EVD testing. After the body was removed, the home was sprayed with disinfectant, and the mattress, clothes, and other personal items used by patient 0 were burned. An attempt was made to identify additional contacts; however, the family was reluctant to cooperate with health officials and reported being angry about the cremation of patient 0 and destruction of property, although these practices were routine at that time for controlling EVD in Monrovia. The family in Monrovia began cooperating with Montserrado County health officials 3 weeks later, on December 18, after learning that 5 family members (patients 1–5) had EVD and after being provided with new mattresses and a small ration of food. At this time, they revealed 2 previously unreported symptomatic family members (patients 7 and 8). As of January 11, 2015, a total of 10 cases were included in this cluster. Eight (80%) patients in this cluster were not identified as contacts before their EVD diagnosis, and 4 (40%) sought care outside the county where they resided (Table; Figure).

**Table:** Characteristics of family members in Ebola cluster, Bong and Montserrado Counties, Liberia, November–December 2014

**Patient no.**	**Relationship to patient 0**	**Age, y/Sex**	**Occupation**	**Date of symptom onset**	Date admitted to ETU*	**Outcome, date**	**City where Ebola exposure likely occurred†**
**0**	–	32/M	Nurse’s aide	Nov 14	–	Died, Nov 24	Monrovia
**1**	Father	78/M	Farmer	Dec 1	Dec 8	Recovered, Dec 24	Monrovia
**2**	Brother	39/M	Auto Mechanic	Dec 9	Dec 10	Recovered, Dec 23	Monrovia
**3**	Niece	15/F	Student	Dec 10	Dec 16	Recovered, Dec 30	Monrovia
**4**	Mother	55/F	Vendor in the market	Dec 15	Dec 17	Died, Dec 22	Monrovia
**5**	Son	3/M	–	Dec 16	Dec 17	Died, Dec 21	Monrovia
**6**	Cousin	29/F	Rubber plantation worker	Dec 16‡	Dec 18	Recovered, Dec 30	Gbarnga
**7**	Sister§	32/F	Vendor in the market	Dec 18	Dec 19	Died, Dec 21	Monrovia
**8**	Brother-in-law#	41/M	Construction worker	Dec 18	Dec 19	Died, Dec 27	Monrovia
**9**	Niece	10/F	Student	Dec 20‡	Dec 20	Recovered, Jan 9 (2015)	Gbarnga

*ETU, Ebola treatment unit. †Gbarnga is located in Bong County; Monrovia is located in Montserrado County. ‡Became symptomatic while under observation at the Bong County Holding Center. §Twin sibling of patient 0. **#Husband of patient 7.**

**Figure:**
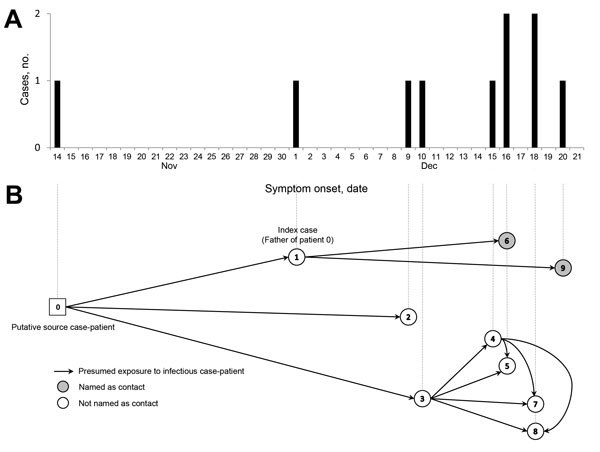
Timeline (A) and transmission diagram (B) of Ebola virus disease cluster, Bong and Montserrado Counties, Liberia, November–December 2014.

## Conclusions

Identifying sources of infection for index patients and tracing contacts are major components of EVD prevention and control efforts (*3*), yet carrying out these policies is challenging when those ill with EVD do not reveal the names of possible sources or contacts who could have been exposed to disease. Detection delays and ineffective contact tracing occurred in this cluster in part because the family believed that the mandatory cremation and property destruction taken as public health actions in Monrovia harmed more than helped. Consequently, some family members sought care in Bong County, riding 4 hours in a taxi from their home in Monrovia, a distance of ≈197 kilometers. Furthermore, family members were reluctant to reveal contact names in Monrovia and initially concealed knowledge of symptomatic persons.

This cluster may have been prevented if patient 0, presumably infected at the clinic where he worked, had been trained in infection control procedures and had access to personal protective equipment. Additional exposures and subsequent infections could have been prevented if he had been identified earlier as a suspected EVD patient, if testing had been performed on his body, if the results had been reported to the family, and if the Monrovia contacts had been followed daily to identify, isolate, and treat symptomatic persons. Had contact tracing identified patients 1–3 as patient 0’s contacts and isolated them immediately after symptoms developed, 6 cases of EVD (in patients 4–9) and 4 deaths (patients 4, 5, 7, and 8) might have been prevented. 

Rapid implementation of contact tracing to prevent disease transmission and increased coordination and communication between jurisdictions are critical to control of EVD. These efforts can identify case-patients who may have entered the community from another jurisdiction (to better understand importation and transmission patterns) and improve case finding and contact tracing to ensure that no cases are missed (*8*,*9*). The effectiveness of these efforts depends on trust between public health officials and the communities they serve.
